# A case control study on family history as a risk factor for herpes zoster and associated outcomes, Beijing, China

**DOI:** 10.1186/s12879-017-2416-7

**Published:** 2017-05-09

**Authors:** Luodan Suo, Li Lu, Juan Li, Mu Sun, Haihong Wang, Xinhui Peng, Fan Yang, Xinghuo Pang, Mona Marin, Chengbin Wang

**Affiliations:** 1 Beijing Center for Disease Control and Prevention, Beijing, China; 2 Xicheng District Center for Disease Control and Prevention, Beijing, China; 3 Changping District Center for Disease Control and Prevention, Beijing, China; 4 Miyun District Center for Disease Control and Prevention, Beijing, China; 5 Centers for Disease Control and Prevention, Atlanta, GA USA

**Keywords:** Herpes zoster, Family history, Postherpetic neuralgia, Recurrent herpes zoster

## Abstract

**Background:**

Hospital-based case control studies have found family history of herpes zoster (HZ) was associated with risk of HZ, but the role of family history is not fully examined for other HZ-associated outcomes such as recurrent HZ, occurrence of postherpetic neuralgia (PHN), and HZ with different pain severities.

**Methods:**

We conducted a population-based matched case control study. HZ cases that occurred during December 1, 2011 to November 30, 2012 were identified by face-to-face interview with all residents of eight selected communities/villages from three districts of Beijing, China. Medical records were reviewed for those who sought healthcare for HZ. For each case-patient, three, age-matched controls (±5 years) without HZ were enrolled from the same community/village of the matched case. Data on family history of HZ were collected by interview and only defined among first-degree relatives.

**Results:**

A total of 227 case-patients and 678 matched controls were enrolled. Case-patients were more likely to report a family history of HZ [odds ratio (OR) =2.4, *P* = 0.002]. Compared with controls, association of family history decreased from HZ with PHN to HZ without PHN (OR = 6.0 and 2.3, respectively; *P* = 0.002 for trend), from recurrent HZ to primary HZ (OR = 9.4 and 2.2, respectively; *P* = 0.005 for trend), and from HZ with moderate or severe pain to HZ with mild or no pain (OR = 3.2 and 0.8, respectively; *P* < 0.001 for trend).

**Conclusions:**

Family history of HZ was associated with HZ occurrence and was more likely in HZ case-patients with PHN, recurrences, and painful HZ.

## Background

Herpes zoster (HZ) (also called shingles) is caused by the reactivation and replication of the latent varicella-zoster virus (VZV) acquired during primary infection (varicella). HZ is usually characterized by unilateral, radicular pain and a vesicular rash generally limited to a single dermatome regulated by the sensory ganglion where the latent VZV reactivated. Though the rash usually heals within two to four weeks, complications from HZ can occur. The most common complication of HZ is postherpetic neuralgia (PHN), generally defined as pain lasting more than 90 days after rash healed. Risk of PHN is very low among persons younger than age 40 but increases with age [1]. HZ frequently occurs on the thoracic and lumbar dermatomes, but the involvement of the eye (HZ ophthalmicus) may lead to serious outcomes and even blindness [2]. Approximately one out three people will develop HZ in their lifetime, and HZ incidence sharply increases after 50 years of age [3, 4].

Waning of cell-mediated immunity (CMI) is the main known determinant for HZ, and factors associated with decline in CMI against VZV have been linked with HZ; these factors include advanced age, immunosuppression, decreased exposure to varicella, and certain chronic conditions [5, 6]. The racial differences in HZ incidence indicate that host-genetic factors might be a risk factor for HZ [7]. Family history, often called the first genetic test, was found to be associated with HZ in several hospital-based, case control studies that also demonstrated a dose response relationship where the dose was number of relatives who have had HZ [8–12]. To date, only one study has examined family history of HZ with risk of developing PHN and found no association [13], and no study has assessed the role of family history for other HZ associated outcomes. To further examine the association of family history with HZ and associated outcomes, we carried out a population-based, matched, case control study in Beijing, China.

## Methods

### Identification and enrollment of study subjects

From December 2012 to March 2013, a cross-sectional, population survey was conducted to assess HZ disease burden in three districts of Beijing area: Xicheng was located in the urban heart of Beijing, Changping located in the north suburb, and Miyun located further northeast in a rural area. A two-step sampling algorithm was used to identify case-patients for the study. First, for each district, each city was categorized according to the varicella incidence during 2007–2011 into one of three groups (high, medium, and low), with a similar number of cities falling within each stratum. One city was randomly selected from each stratum, therefore, nine cities were sampled; eight agreed to participate. Second, one community/village was randomly selected from each of the eight participating cities. All residents who had lived in the area for at least six months during December 1, 2011 to November 30, 2012 were eligible to be study participants. All eligible participants of the community/village were contacted and interviewed face-to-face until reaching the target of 15,000 participants per community/village. The adjacent community/village would be enrolled if the first community/village had less than 15,000 participants enrolled in each city. An HZ case was defined as: 1) a diagnosis of HZ made by a medical provider (confirmed by review of medical records), or 2) if care was not sought, self-diagnosis of HZ based on the presence of clustered blisters on one dermatome with pain, itch, and hypersensitivity, during December 1, 2011 and November 30, 2012.

For each identified case-patient, three, age-matched (±5 years) controls were randomly selected from the residents who had never had HZ and who lived in the same community or village as the case-patient.

### Data collection

Both case-patients and controls were interviewed using a standard form that collected data on sociodemographic characteristics and chronic conditions. Further information on HZ characteristics was collected from the case-patients, including the date of rash onset, whether they had a prior episode of HZ (recurrent HZ), pain severity (no or mild pain, moderate pain with little limitation of daily activities, and severe pain with important limitation of daily activities), and duration of pain after rash healed. PHN was defined as pain persisting 90 days or more after HZ rash healed [14]. Data on family history of HZ were collected for both case-patients and controls and included whether the subjects were aware of any family members/relatives who ever had HZ and the relationship to the subject interviewed, which was used to determine the degree of blood relation (first degree vs. non-first degree relative).

### Statistical analyses

Conditional logistic regression was used to assess the association of family history with HZ. We also examined the association of family history with HZ associated outcomes such as recurrent or primary HZ, HZ with different pain severity, or HZ with and without PHN. Since HZ is more likely to occur in females, the associations of family history with HZ were further examined in males and females, respectively. To maximize the accuracy of family history data, we restricted the analysis to first degree relatives only (defined as parents, offspring, and siblings) considering that reports of HZ occurrence among the first degree relatives were less subject to recall bias (because of more and closer interactions) and to information bias (subjects were more likely to be aware of occurrence of HZ among first degree relatives). Subjects who reported only a non-first degree relative with HZ were classified as not having a family history of HZ (6 case-patients and 6 controls).

The distribution of categorical or continuous sociodemographic factors between cases and controls were examined with Mantel-Haenszel chi-square or student t-test, respectively. The trend test was used to assess the association of family history with different HZ outcomes with multiple levels (e.g., dose response), such as primary and recurrent HZ, HZ with and without PHN, and HZ with different pain severities. All analyses were performed with SAS version 9.3 (SAS Inc., Cary, NC).

Beijing Center for Disease Control and Prevention Ethics Committee on Human Subjects reviewed and approved the project.

## Results

### Characteristics of study subjects

A total of 905 subjects (227 case-patients and 678 age matched controls) were enrolled. The median age of subjects was 54.4 years (range: 11.1–85.5 years) and more than half were female (486, 53.7%). The majority of subjects were local residents (799, 88.4%), had less than high school education (604, 66.7%), were farmers (483, 53.4%) and had no underlying medical conditions (718, 79.3%). Among those with underlying medical conditions, hypertension was the most common condition (108, 57.8%), followed by diabetes (43, 23.0%) and cardiovascular disease (18, 9.6%). One subject had an organ transplant, one had uremia, one had cancer, and one was on immunosuppressive medication. There were no differences between case-patients and controls with respect to sociodemographic and medical characteristics (Table 1). For HZ case-patients, the median duration of pain was 20 days (range: 1–365 days), and the majority of HZ patients had moderate or severe pain (160, 70.5%); 11 (4.8%) developed PHN. HZ case-patients with PHN did not differ from those without PHN by age (*P* = 0.13), sex (*P* = 0.35), or underlying medical conditions (*P* = 1.0). A total of 26 subjects (11.5%) experienced recurrent HZ. There was no difference between case-patients with primary and recurrent HZ by age (*P* = 0.40), sex (*P* = 0.28), or underlying medical conditions (*P* = 0.15). A total of 18 (7.9%) HZ case-patients were self-diagnosed with no difference between self-diagnosed HZ and HZ diagnosed by medical providers by age (*P* = 0.17), sex (*P* = 0.62), or underlying medical conditions (*P* = 0.38).

**Table 1:** Distribution of Sociodemographic Factors between the Herpes Zoster Case-Patients and Controls

	Case-Patients, n (%)	Controls, n (%)	
	*N* = 227	*N* = 678	*P* value
Sex			0.53
Male	101 (44.5)	318 (46.9)	
Female	126 (55.5)	360 (53.1)	
Age (years)			0.92
< 40	47 (20.7)	140 (20.7)	
40–49	45 (19.8)	138 (20.4)	
50–59	55 (24.2)	173 (25.5)	
60–69	54 (23.8)	142 (20.9)	
≥ 70	26 (11.5)	85 (12.5)	
Education			0.17
Elementary school or less	78 (34.3)	233 (34.3)	
Middle school	62 (27.3)	231 (34.1)	
High school	51 (22.5)	133 (19.6)	
College and above	36 (15.9)	81 (12.0)	
Residency			0.93
Local resident	201 (88.6)	598 (88.3)	
Mobile population	26 (11.5)	79 (11.7)	
Nationality			0.16
Han	215 (94.7)	655 (96.8)	
Other	12 (5.3)	22 (3.3)	
Profession			0.45
Farmer	117 (51.5)	366 (54.0)	
Worker	31 (13.7)	63 (9.3)	
Teacher	4 (1.8)	11 (1.6)	
Healthcare worker	0 (0.0)	7 (1.0)	
Government employee	9 (4.0)	16 (2.4)	
Retired	25 (11.00	79 (11.7)	
Student	1 (0.4)	4 (0.6)	
Unemployed	12 (5.3)	44 (6.5)	
Other	28 (12.3)	88 (13.0)	
Underlying medical condition			0.25
Yes	53 (23.4)	134 (19.8)	
No	174 (76.7)	544 (80.2)	

### Family history and HZ and associated outcomes

Overall, HZ case-patients were 2.4 times more likely to report a family history of HZ (among the first degree relatives) than their matched controls (*P* = 0.002) (Table 2). The strength of association between family history and risk of HZ in males was almost twice that of females (3.3 vs. 1.7) (Table 3). There were only two case-patients with PHN and family history of HZ. Compared with the controls, case-patients with PHN were six times more likely to report a family history of HZ, though not statistically significant (*P* = 0.14), and case-patients with HZ without PHN were 2.3 times more likely to report family history (*P* = 0.006). In addition, case-patients with recurrent HZ were nine times more likely to report a family history (*P* = 0.05), while case-patients with primary HZ were around two times more likely to report a family history when compared with controls (*P* = 0.01 and *P* < 0.001 trend). Furthermore, compared with controls, HZ case-patients with moderate or severe pain were three times more likely to report a family history of HZ (*P* < 0.001), while no difference was found for case-patients with mild or no pain (*P* = 0.72) (Table 2). We did not examine the association of having more than one relative with HZ due to lack of data; there were only one case-patient and four controls who reported family history of HZ in more than one relative.

**Table 2:** Association of Family History with Herpes Zoster with Different Characteristics

HZ with different characteristics	Family history^a^		Odds ratio (95% confidence interval)		*P* value for trend test
	Yes, n (%)	No, n (%)		*P* value	
Controls	33 (4.9)	645 (95.1)	Reference		
All HZ case-patients	24 (10.6)	203 (89.4)	2.4 (1.4–4.3)	0.002	NA
HZ with PHN	2 (18.2)	9 (81.8)	6.0 (0.5–66.2)	0.14	0.002
HZ without PHN	22 (10.2)	194 (89.8)	2.3 (1.3–4.2)	0.006	
Recurrent HZ	4 (15.4)	22 (84.6)	9.4 (1.0–88.5)	0.05	0.005
Primary HZ	20 (10.0)	181 (90.0)	2.2 (1.2–3.9)	0.01	
HZ with moderate/severe pain	21 (13.1)	139 (86.9)	3.2 (1.7–6.0)	<0.001	<0.001
HZ with mild or no pain	3 (4.5)	64 (95.5)	0.8 (0.2–3.3)	0.72	

*HZ* herpes zoster; ^a^defined as history of HZ among first degree relatives (i.e., parents, offspring, siblings)

**Table 3:** Different Strength of Association between Family History and Herpes Zoster by Sex^a^

	Family history		Odds ratio (95% confidence interval)	
	Yes, n (%)	No, n (%)		*P* value
Male participants				
HZ case-patients	11 (64.7)	90 (38.1)	3.3 (1.1–10.1)	0.04
Controls	6 (35.3)	146 (61.9)	Reference	
Female participants				
HZ case-patients	13 (46.4)	113 (36.8)	1.7 (0.7–4.0)	0.22
Controls	15 (53.6)	194 (63.2)	Reference	

*HZ* herpes zoster; ^a^controls with discordant sex from the matched case were removed from the analysis, leading to reduced number of controls included in this stratified analysis

## Discussion

Our study provides further evidence that family history is associated with HZ. Additionally, we found that association of family history of HZ was stronger among recurrent HZ, HZ with PHN, HZ with moderate or severe pain, and males.

The strength of the association between family history and HZ in our study (OR = 2.4) is in the range of reported strength of association between family history and HZ from five other case control studies, three from the US and one from Iran and France (OR ranging from 1.7 to 6.6), even though hospital controls were used for all these five studies [8–12] Some of these studies examined the association of first degree relatives and found the strength of association ranged from 1.9 to 4.9 [8–10, 12], but they were inconsistent on the association of non-first degree relative with HZ, range from no association [12] to strong association (OR = 4.3 and 4.8, respectively) [9, 10]. Albeit consensus has been reached on the association of family history and HZ [15], no study examined the strength of association in different races (as an indicator for differences in genetic predisposition), even though racial differences in HZ incidence are well documented [7]. More studies are needed to examine the effect of family history in HZ by race.

We also found a family history of HZ was more strongly associated with recurrent HZ than primary HZ. This finding may indicate that those with recurrent HZ have a higher genetic predisposition for HZ. There are reports on genetic susceptibility for other recurrent infections such as upper respiratory tract infections and genital chlamydia infection [16, 17], in which the same immunogenetic factors as reported for HZ occurrence (IL-10 polymorphism) were implicated [18, 19]. The proportion of recurrent HZ among case-patients in our study were similar to reoccurrence in other studies that also used self-reported HZ [20].

In addition, we found that family history was more strongly associated with HZ with moderate or severe pain than with mild or no pain. HZ-related pain intensity was associated with the duration of HZ-related pain [21, 22], with longer duration of HZ-related pain linked to increased risk of recurrent HZ [23]. As in a previous report [13], we did not detect a statistically significant association between family history of HZ and PHN, but the strong association we found amongst the small number of PHN case-patients in our study warrant further study with a sufficient sample size, not to mention the scarcity of assessment on family history in PHN. In addition, we found effect modification by sex on family history association with HZ, with family history having a stronger effect on HZ risk in males [24]. Further studies are needed to verify the effect of sex on the association of family history and HZ risk.

Several limitations should be taken into account when interpreting the results of our study. Recall bias regarding family members with HZ might be differential between cases and controls. We attempted to minimize the recall bias by restricting the analysis to first-degree relatives; however, this bias is hard to eliminate. We included self-diagnosed HZ which could potentially increase the misclassification of HZ and weaken the association of family history with HZ risk. However, the proportion of self-diagnosed HZ was low and it has been found that self-reported HZ had high consistency with physician diagnosis in other populations [25]. Another limitation of using only first-degree relatives to define family history was that it reduced the number of relatives with family history, and made us unable to assess a dose-response relationship by the number of family members with a family history of HZ as in prior reports.

## Conclusions

In summary, our study found that family history of HZ was associated with HZ occurrence. We also found that a family history of HZ was more strongly associated with recurrent HZ than primary HZ and HZ with moderate or severe pain than with no or mild pain. Given the availability of effective HZ vaccines [21, 26], vaccination of persons with risk factors for HZ or more severe HZ may be an important approach to either prevent HZ or limit suffering in these patients.

## Abbreviations

CMI: Cell-mediated immunity
HZ: Herpes zoster
OR: Odds ratio
PHN: Postherpetic neuralgia
VZV: Varicella zoster virus

## References

[1] Yawn BP, Gilden D (2013). The global epidemiology of herpes zoster. Neurology.

[2] Grose C (2010). Stroke after varicella and zoster ophthalmicus: another indication for treatment and immunization. Pediatr Infect Dis J.

[3] Oxman MN (2010). Zoster vaccine: current status and future prospects. Clin Infect Dis.

[4] Yawn BP, Saddier P, Wollan PC, St Sauver JL, Kurland MJ, Sy LS (2007). A population-based study of the incidence and complication rates of herpes zoster before zoster vaccine introduction. Mayo Clin Proc.

[5] Johnson RW, Alvarez-Pasquin MJ, Bijl M, Franco E, Gaillat J, Clara JG, Labetoulle M, Michel JP, Naldi L, Sanmarti LS (2015). Herpes zoster epidemiology, management, and disease and economic burden in Europe: a multidisciplinary perspective. Ther Adv Vaccines.

[6] Forbes HJ, Thomas SL, Smeeth L, Clayton T, Farmer R, Bhaskaran K, Langan SM (2016). A systematic review and meta-analysis of risk factors for postherpetic neuralgia. Pain.

[7] Schmader K, George LK, Burchett BM, Hamilton JD, Pieper CF (1998). Race and stress in the incidence of herpes zoster in older adults. J Am Geriatr Soc.

[8] Hernandez PO, Javed S, Mendoza N, Lapolla W, Hicks LD, Tyring SK (2011). Family history and herpes zoster risk in the era of shingles vaccination. J Clin Virol.

[9] Hicks LD, Cook-Norris RH, Mendoza N, Madkan V, Arora A, Tyring SK (2008). Family history as a risk factor for herpes zoster: a case-control study. Arch Dermatol.

[10] Ansar A, Farshchian M, Ghasemzadeh M, Sobhan MR (2014). Association between family history and herpes zoster: a case-control study. J Res Health Sci.

[11] Lasserre A, Blaizeau F, Gorwood P, Bloch K, Chauvin P, Liard F, Blanchon T, Hanslik T (2012). Herpes zoster: family history and psychological stress-case-control study. J Clin Virol.

[12] Marin M, Harpaz R, Zhang J, Wollan P, Bialek S, Yawn B (2016). Risk factors for herpes zoster among adults. Open Forum Infect Dis.

[13] Gatti A, Pica F, Boccia MT, De Antoni F, Sabato AF, Volpi A (2010). No evidence of family history as a risk factor for herpes zoster in patients with post-herpetic neuralgia. J Med Virol.

[14] Sampathkumar P, Drage LA, Martin DP (2009). Herpes zoster (shingles) and postherpetic neuralgia. Mayo Clin Proc.

[15] Lai YC, Yew YW (2016). Risk of herpes zoster and family history: a meta-analysis of case-control studies. Indian J Dermatol.

[16] Zehsaz F, Farhangi N, Monfaredan A, Tabatabaei Seyed M (2015). IL-10 G-1082A gene polymorphism and susceptibility to upper respiratory tract infection among endurance athletes. J Sports Med Phys Fitness.

[17] Wang C, Tang J, Geisler WM, Crowley-Nowick PA, Wilson CM, Kaslow RA (2005). Human leukocyte antigen and cytokine gene variants as predictors of recurrent Chlamydia trachomatis infection in high-risk adolescents. J Infect Dis.

[18] Cho JW, Shin DH, Lee KS (2007). Polymorphism of the IL-10 gene is associated with susceptibility to herpes zoster in Korea. J Dermatol Sci.

[19] Haanpaa M, Nurmikko T, Hurme M (2002). Polymorphism of the IL-10 gene is associated with susceptibility to herpes zoster. Scand J Infect Dis.

[20] Bowsher D (1999). The lifetime occurrence of herpes zoster and prevalence of post-herpetic neuralgia: a retrospective survey in an elderly population. Eur J Pain.

[21] Oxman MN, Levin MJ, Johnson GR, Schmader KE, Straus SE, Gelb LD, Arbeit RD, Simberkoff MS, Gershon AA, Davis LE (2005). A vaccine to prevent herpes zoster and postherpetic neuralgia in older adults. N Engl J Med.

[22] Dworkin RH, Johnson RW, Breuer J, Gnann JW, Levin MJ, Backonja M, Betts RF, Gershon AA, Haanpaa ML, McKendrick MW (2007). Recommendations for the management of herpes zoster. Clin Infect Dis.

[23] Yawn BP, Wollan PC, Kurland MJ, St Sauver JL, Saddier P (2011). Herpes zoster recurrences more frequent than previously reported. Mayo Clin Proc.

[24] Opstelten W, Van Essen GA, Schellevis F, Verheij TJ, Moons KG (2006). Gender as an independent risk factor for herpes zoster: a population-based prospective study. Ann Epidemiol.

[25] Schmader K, George LK, Newton R, Hamilton JD (1994). The accuracy of self-report of herpes zoster. J Clin Epidemiol.

[26] Lal H, Cunningham AL, Godeaux O, Chlibek R, Diez-Domingo J, Hwang SJ, Levin MJ, McElhaney JE, Poder A, Puig-Barbera J (2015). Efficacy of an adjuvanted herpes zoster subunit vaccine in older adults. N Engl J Med.

